# New perspectives in signaling mediated by receptors coupled to stimulatory G protein: the emerging significance of cAMP eﬄux and extracellular cAMP-adenosine pathway

**DOI:** 10.3389/fphar.2015.00058

**Published:** 2015-03-25

**Authors:** Rosely O. Godinho, Thiago Duarte, Enio S. A. Pacini

**Affiliations:** Disciplina Farmacologia Celular, Departamento de Farmacologia, Escola Paulista de Medicina, Universidade Federal de São PauloSão Paulo, Brazil

**Keywords:** G protein-coupled receptors, cyclic AMP, cAMP eﬄux, adenosine, adenosine receptors, ABC transporters, ecto-phosphodiesterase, ecto-5′-nucleotidase

## Abstract

G protein-coupled receptors (GPCRs) linked to stimulatory G (Gs) proteins (GsPCRs) mediate increases in intracellular cyclic AMP as consequence of activation of nine adenylyl cyclases , which differ considerably in their cellular distribution and activation mechanisms. Once produced, cyclic AMP may act via distinct intracellular signaling effectors such as protein kinase A and the exchange proteins activated by cAMP (Epacs). More recently, attention has been focused on the eﬄux of cAMP through a specific transport system named multidrug resistance proteins that belongs to the ATP-binding cassette transporter superfamily. Outside the cell, cAMP is metabolized into adenosine, which is able to activate four distinct subtypes of adenosine receptors, members of the GPCR family: A_1_, A_2A_, A_2B_, and A_3_. Taking into account that this phenomenon occurs in numerous cell types, as consequence of GsPCR activation and increment in intracellular cAMP levels, in this review, we will discuss the impact of cAMP eﬄux and the extracellular cAMP-adenosine pathway on the regulation of GsPCR-induced cell response.

Seven transmembrane receptors are able to transmit extracellular signals into the intracellular compartment via activation of heterotrimeric G proteins, which consist of the guanine nucleotide-binding Gα subunit and the dimeric βγ subunits (Pierce et al., 2002). These G protein-coupled receptors (GPCRs) are ubiquitously distributed and precisely regulate a myriad of intracellular processes upon agonist stimulation. GPCRs may also activate β-arrestin-dependent signaling, which functions as an adaptor protein that regulates GPCR signaling and trafficking, having the ability to activate intracellular pathways independently of G proteins (DeWire et al., 2007).

The membrane-associated G proteins can be divided into four families according to the primary sequence similarity of Gα subunits: Gαs (α_s_, α_olf_), Gαi (α_i1_–α_i3_, α_t_, α_o1_–α_o2_, α_ζ_), Gα_q/11_ (α_q_, α_11_, α_14_–α_16_), Gα_12_ (α_12_, α_13_). The sequence homologies also define the Gα subunit coupling to downstream effector molecules, such as phospholipase C-β, adenylyl cyclase (AC), RhoGEFs and/or ion channels (Moreira, 2014).

Conformational changes of GPCR, induced by extracellular agonist binding, reduces the affinity of GDP to Gα subunit, resulting in sequential dissociation of GDP-Gα complex, interaction of GTP with Gα, and dissociation of heterotrimeric complex Gα–βγ into Gα subunit and βγ dimmer (Pierce et al., 2002). Focusing on activation of GPCRs linked to stimulatory G (Gs) proteins (GsPCR), the GTP-Gαs complex is able to interact and activate all available membrane bound AC isoforms (AC1–AC9), increasing the intracellular generation of adenosine 3′,5′-cyclic monophosphate (3′,5′-cAMP or cAMP). Conversely, by activating inhibitory G (Gi) protein, agonists of GiPCR elicit the selective inhibition of AC1, AC5, and AC6, reducing the intracellular cAMP content(Sadana and Dessauer, 2009).

Cyclic AMP was first described by Rall et al. (1957) who showed that sympathomimetic amines and glucagon were able to induce the synthesis of a heat-stable factor formed by particulate fractions of liver homogenates in the presence of ATP and Mg^2+^. Identified as adenine ribonucleotide, the compound was identical to the cAMP described by Cook et al. (1957) at the same year. Since then, intracellular signaling through cAMP has been described in virtually every cell (Antoni, 2012). The protein kinase A (PKA), a serine/threonine kinase ubiquitously expressed in mammal tissues, was the first cAMP downstream target to be identified (Walsh et al., 1968). Formed by two regulatory and two catalytic subunits, activation of PKA involves the cooperative binding of four cAMP molecules to two regulatory subunits, leading the catalytic subunits free to phosphorylate target proteins. Intracellular cAMP is also able to directly modulate ion channels (Bradley et al., 2005), such as the hyperpolarization-activated cyclic nucleotide-gated channels (HCN channels) expressed in the cardiac sinoatrial node (Larsson, 2010). Additionally, exchange proteins directly activated by cAMP (Epac) explained various effects of cAMP that could not be attributed to the PKA or cAMP-gated ion channels (Gloerich and Bos, 2010).

## Phosphodiesterases, MRP/ABCC Transporters and cAMP Eﬄux

The fine regulation of intracellular cAMP signaling is made by phosphodiesterases (PDEs), enzymes that catalyze the hydrolysis of cAMP into AMP. Actually, the superfamily of PDE enzymes is comprised of 11 families, namely PDE1–PDE11. While PDE4, PDE7, and PDE8 are specific for cAMP, PDE5–PDE6, and PDE9 are specific for guanosine 3′,5′-cyclic monophosphate (cGMP) and PDE1–PDE3 and PDE10–PDE11 hydrolyze both cAMP and cGMP (Bender and Beavo, 2006; Francis et al., 2011). By hydrolyzing the cyclic nucleotide monophosphates, PDEs regulate several important physiological processes, such as vascular resistance, cardiac output, visceral motility, immune response, inflammation, neuroplasticity, vision, and reproduction (Azevedo et al., 2014). Therefore, altered expression of PDE or changes in the enzyme activity have been associated with a number of pathological conditions, including erectile dysfunction, pulmonary hypertension, acute refractory cardiac failure, chronic obstructive pulmonary disease and various types of cancer (Azevedo et al., 2014).

In addition to PDE, the intracellular concentration of cAMP is regulated by its eﬄux into the extracellular space through a specific transport system named multidrug resistance proteins (MRP; Cheepala et al., 2013) that belongs to the ATP-binding cassette (ABC) transporter superfamily (subfamily C). Three of them (MRP4/ABCC4, MRP5/ABCC5, and MRP8/ABCC11) have the ability to actively extrude cAMP and cGMP from the cell (Kruh and Belinsky, 2003) with different kinetic parameters (Km values in the μM range; Jedlitschky et al., 2000; Chen et al., 2001; Guo et al., 2003; Sager and Ravna, 2009).

The MRP-mediated cAMP eﬄux has been shown in many cell types, including rodent skeletal muscle (Godinho and Costa, 2003), human platelets (Jedlitschky et al., 2004) vascular smooth muscle cells (Sassi et al., 2008; Cheng et al., 2010), pulmonary arteries (Hara et al., 2011) and cardiac myocytes (Sassi et al., 2011). Therefore, it was proposed that by working synergistically with PDE enzymes, MRP-mediated cAMP eﬄux would preserve the cell from excessive levels of intracellular cAMP (Jedlitschky et al., 2000; Chen et al., 2001). Although reasonable, this premise was not consistent with the high energetic cost of pumping cAMP out of cell (Godinho and Costa, 2003; Chiavegatti et al., 2008). Thus, it was hypothesized that cAMP might also function as an extracellular signaling molecule (Jackson and Raghvendra, 2004). In fact, since the middle 1960s, extracellular effects of cAMP have been reported in different mammal tissues and organs. For example, intravenous infusion of cAMP is known to produce a wide range of responses, including increased adrenal corticosterone secretion in hypophysectomized rats (Imura et al., 1965), increment in heart rate, cardiac output, and blood glucose, reduction of blood pressure (Levine and Vogel, 1966) and increment in calcium and phosphate plasma concentration (Rasmussen et al., 1968). cAMP infusion effects seem to be exclusively extracellular since most cells are impermeable to cAMP (Robison et al., 1965).

## Ecto-Phosphodiesterases, Ecto-Nucleotidases, and the Extracellular cAMP-Adenosine Pathway

The idea of an extracellular role for cAMP gained strength in the late 1990s when it was established that extracellular cAMP could be sequentially metabolized into AMP and adenosine, a phenomenon named “*extracellular cAMP-adenosine pathway*” (Mi and Jackson, 1998). Actually, the existence of ecto-enzymes with PDE activity was first demonstrated in frog skeletal muscle by Woo and Manery (1973), who showed extracellular generation of [^14^C]AMP as consequence of incubation of intact posterior leg muscles with [^14^C]cAMP. The degradation of cAMP was markedly inhibited by theophylline, suggesting the presence of cAMP-phosphodiesterase activity at the muscle fiber surface (Woo and Manery, 1973). Later, ecto-phosphodiesterase activity was found in rodent liver cells (Smoake et al., 1981), vascular smooth muscles (Dubey et al., 1996; Jackson et al., 1997), adipocytes (Strouch et al., 2005), skeletal muscle (Chiavegatti et al., 2008), rat ileum (Giron et al., 2008), astrocytes and microglia (Verrier et al., 2011) and many other cells.

In those cells, following the extracellular degradation of 3′,5′-cAMP by ecto-PDE, 5′-AMP is rapidly hydrolyzed to adenosine by membrane-bound ecto-5′-nucleotidases (EC 3.1.3.5), also known as CD73. The ecto-5′-nucleotidase is a 70-kDa cell surface glycoprotein found in most tissues that functions as the major enzymatic source of interstitial adenosine (Colgan et al., 2006). Although ecto-5′-nucleotidases also hydrolyzes other ribo- and deoxyribonucleoside 5′-monophosphates including cytidine-, uridine-, inosine-, and guanosine-5′-monophosphates, AMP is the most effectively hydrolyzed nucleotide (Zimmermann et al., 2012). Thus, by hydrolyzing 5′-AMP derived from either ATP or cAMP, ecto-5′-nucleotidases act as a limiting point for the extracellular provision of adenosine and, as consequence, for activation of adenosine receptors (ARs).

In addition to ecto-5′-nucleotidases, other three types of surface-located ecto-nucleotidases are involved in the extracellular nucleosides degradation and, consequently, in the extracellular adenosine formation: ecto-nucleoside triphosphate diphosphohydrolases (EC 3.6.1.5), ecto-nucleotide pyrophosphatase/PDEs (EC 3.6.1.9 and EC 3.1.4.1) and alkaline phosphatases (APs; EC 3.1.3.1). While ecto-5′-nucleotidases is nucleotide-specific, ecto-nucleotide pyrophosphatase/PDEs and ecto-nucleoside triphosphate diphosphohydrolases hydrolyze both ATP and ADP (Zimmermann et al., 2012).

## Adenosine Receptors and the Extracellular cAMP-Adenosine Pathway

The extracellular degradation of cAMP allows the activation of membrane associated ARs by its metabolite adenosine. Pharmacologically, four distinct subtypes of ARs, members of the GPCR family, are described: A_1_, A_2A_, A_2B,_ and A_3_ (Fredholm et al., 2011). These receptors have a very broad tissue distribution, performing relevant physiological effects in central nervous system (Ribeiro et al., 2002; Wei et al., 2011), cardiovascular (Headrick et al., 2013; Idzko et al., 2014a) and musculoskeletal systems (Burnstock et al., 2013) and many other tissues (Vallon et al., 2006; Sheth et al., 2014). Activation of adenosine is also involved in pathophysiological conditions such as cancer, inflammation (Antonioli et al., 2013; Idzko et al., 2014b) and neurodegenerative diseases, with potential therapeutic implications for Parkinson’s and Alzheimer’s diseases (Ribeiro et al., 2002).

**FIGURE 1:**
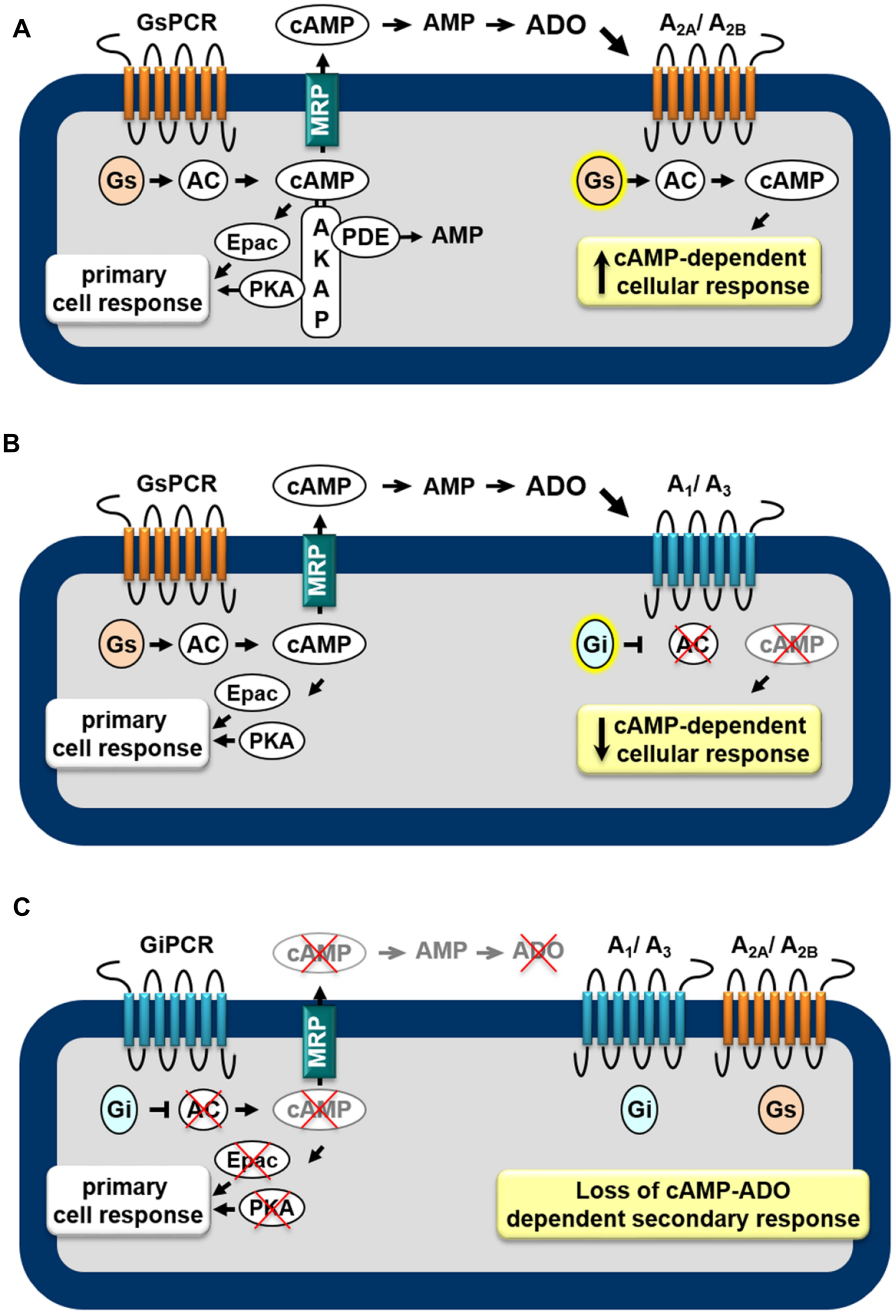
**Feedback mechanisms mediated by the extracellular cAMP–adenosine cascade.** Activation of receptors coupled to Gs protein (GsPCR) leads to stimulation of adenylyl cyclase (AC) and increased generation of cAMP, which may elicit localized cellular response via activation of effectors such as PKA or Epac **(A,B)**, which are organized in microdomains via anchoring proteins, such as AKAP. cAMP may be hydrolyzed by intracellular phosphodiesterases (PDEs) or may leave the cell via multidrug resistance proteins (MRPs). Outside the cell, ecto-PDE and ecto-5′nucleotidase sequentially convert cAMP to AMP and adenosine (ADO), which may activate Gs-coupled A_2A_/A_2B_ **(A)** or Gi-coupled A_1_/A_3_ **(B)** receptors, increasing or attenuating cAMP production, respectively. In addition, activation of receptors coupled to Gi protein (GiPCR) results in inhibition of Gi-sensitive ACs reducing both generation and eﬄux of cAMP **(C)**, with consequent loss of autocrine/paracrine feedback signaling of extracellular cAMP.

AR subtypes exhibit different affinities for the endogenous agonist (Muller and Jacobson, 2011): A_1_, A_2A,_ and A_3_ display high to moderate affinity (Ki = 100, 310, and 290 nM, respectively) to adenosine whereas A_2B_ has low affinity (Ki = 15 μM). Furthermore, distinct signaling transduction pathways are mobilized by each AR, leading to diverse cellular effects. The A_1_ and A_3_ are preferentially coupled to the Gi/o family of G-proteins whereas the A_2A_ and A_2B_ couple to Gs proteins (Trincavelli et al., 2010). Thus, as shown in **Figure 1**, depending on the receptor subtypes expressed in the cell, the *extracellular cAMP-adenosine pathway* will be able to increase (**Figure 1A**) or attenuate (**Figure 1B**) the intracellular cAMP production, by activating or inhibiting ACs, via Gs and Gi proteins, respectively. Therefore, the final biological effect of the *extracellular cAMP-adenosine pathway* will depend on AR subtype expressed by the target cell and on the amount of cAMP pumped out of the cell.

In fact, the extracellular cAMP-adenosine signaling can be even more complex, as in addition to classical Gs and Gi pathways, AR subtypes can also activate Gq protein (A_2B_ and A_3_), phospholipase C-β (A_1_, A_2B_, and A_3_), PKC (A_2B_ and A_3_), K_ATP_ channel (A_3_), and ERK1/2 (A_1_, A_2A_/A_2B_, and A_3_) p38 (A_1_ and A_2B_) MAPK cascade (Feoktistov et al., 1999; Schulte and Fredholm, 2003), which in turn are able to modulate AC activation (Sadana and Dessauer, 2009).

## Activation of GsPCR and the Extracellular cAMP-Adenosine Pathway

Taking into account that cAMP may function as an extracellular signaling molecule, it is possible to assume that, by stimulating AC enzymes, all GsPCRs may have significant impact on cAMP eﬄux and extracellular cAMP-adenosine signaling. Actually, by studying the contribution of cAMP eﬄux and the *extracellular cAMP-adenosine pathway* on skeletal muscle physiology, we showed that cAMP egress induced by activation of GsPCR, such as β-adrenoceptors and calcitonin gene-related peptide receptors, is proportional to intracellular levels of cAMP produced (Godinho and Costa, 2003; Chiavegatti et al., 2008). Of more importance, cAMP eﬄux and subsequent extracellular generation of adenosine elicited by β_2_-adrenoceptor agonists results in a delayed attenuation of β_2_-adrenoceptor-induced positive inotropic effect, via activation of A_1_ ARs (Duarte et al., 2012), as schematically shown in **Figure 1B**.

The correlation of GsPCR activation, cAMP eﬄux, and the *extracellular cAMP-adenosine pathway* has been described in many other cells. For example, the β-adrenoceptor agonist isoproterenol stimulates cAMP secretion from rat cultured glioma cells (Doore et al., 1975) and turkey erythrocytes (Rindler et al., 1978). In mouse adrenocortical tumor cell line, the intracellular cAMP formed in response to ACTH appeared extracellularly in few minutes (Schimmer and Zimmerman, 1976). In healthy volunteers, infusion of glucagon after hepatic venous catheterization was associated with a marked increase in both the splanchnic cAMP production and in the arterial cyclic nucleotide levels (Grill et al., 1979). In addition, while activation of D_1_ dopamine receptor stimulates cAMP eﬄux from rat neostriatal cells (Plantie et al., 1983), vasoactive intestinal peptide and cholecystokinin-8 cause a significant increase in cAMP eﬄux from rat striatal slices (O’Shaughnessy et al., 1987). Many other GsPCR agonists are able to stimulate cAMP eﬄux, such as corticotropin-releasing hormone and melanocortin (de Koning et al., 1992), vasopressin (Tyagi et al., 1998) and pituitary AC activating polypeptide (Cui et al., 2000). Taking into account that cAMP eﬄux depends on increased intracellular generation of cyclic nucleotide it is possible to presume that cAMP export could be ubiquitously elicited by activation of every GsPCR.

Conversely, it is also plausible to postulate that by inhibiting Gαi-sensitive ACs and reducing intracellular cAMP generation, receptors linked to Gi protein (GiPCR) would be able to negatively modulate cAMP eﬄux. For instance, cAMP egress from rat neostriatal slices induced by activation of Gs-linked D-1 dopamine receptors is reduced by agonists of D_2_ receptor agonists (Stoof and Kebabian, 1981), μ- or δ opioid receptors (Schoffelmeer et al., 1985) and M_2_ and M_4_ muscarinic receptors (Schoffelmeer et al., 1988), known to be coupled to Gi-protein. Indeed, several pheromones and fatty acids, components of membrane phospholipids, also inhibit cAMP export (Kanter et al., 1989) by activating free fatty acid receptors, such as FFA5, linked to Gi/o protein (Wang et al., 2006). Thus, inhibition of cAMP egress as consequence of inhibition of cAMP production may provide an additional mechanism by which GiPCR agonists influence the cAMP signaling pathway, as illustrated in **Figure 1C**. **Table 1** presents a list of GPCR receptors linked to Gs and Gi protein (Alexander et al., 2013) that, by modulating intracellular generation of cAMP, may function as regulators of cAMP eﬄux and *extracellular cAMP adenosine pathway*.

**Table 1:** Receptors coupled to stimulatory (Gs) and inhibitory (Gi) G proteins th

	Gα subunits	
	**Receptors coupled to inhibitory G protein (Gαi)**
	**Orphans:** GPR22, GPR34, GPR37, GPR84,1 MRGPRD, GPR33, GPR37L1, OPN5	**Hydroxycarboxylic acid:** HCA_1_, HCA_2_, HCA_3_
	**Acetylcholine (Muscarinic):** M_2_, M_4_	**5-Hydroxytryptamine:** 5-HT_1A_, 5-HT_1B_, 5-HT_1D_–5-HT_1F_, 5-Ht_5A_
	**Adenosine:** A_1_, A_3_	**Leukotriene/lipoxin/oxoeicosanoids:** BLT_1_–BLT_2_, FPR_2_/ALX, OXE
	**Adrenoceptor (α):** α_2A_, α_2B_, α_2C_	**Lysophospholipid (LPA):** LPA_1_–LPA_4_
	**Angiotensin:** AT_2_	**Sphingosine 1-phosphate (S1P):** S1P_1_, S1P_3_–S1P_5_
	**Apelin:** HGNC	**Melanin-concentrating hormone (MCH):** MCH_1_
	**Calcium sensing:** CaS	**Melatonin:** MT_1_–MT_2_
	**Cannabinoid:** CB_1_, CB_2_	**Metabotropic glutamate (mGlu):** mGlu_2_–mGlu_4_; mGlu_6_–mGlu_8_
	**Chemokine:** CCR_1_–CCR_10_, CXCR_1_–CXCR_6_, CX_3_CR1, XCR1	**Neuropeptide FF/neuropeptide AF:** NPFF2
	**Complement peptide**: C3a, C5a_1_	**Neuropeptide W/neuropeptide B:** NPBW1–NPBW2
	**Dopamine:** D_2_, D_3_, D_4_	**Neuropeptide Y:** Y_1_–Y_2_, Y_4_–Y_6_
	**Endothelin:** ET_B_	**Opioid:** δ, κ, μ, NOP
	**Estrogen (G protein-coupled):** GPER	**Purinergic P2Y:** P2Y_12_–P2Y_13_
	**Formylpeptide:** FPR1, FPR2/ALX	**Peptide P518:** QRFP
	**Free fatty acid:** FFA3, FFA2, FFA5	**Platelet-activating factor:** PAF
	**Gaba:** Gaba_B_	**Prostanoid:** DP_2_, EP_3_
	**Galanin:** GAL_1_, GAL_2_, GAL_3_	**Proteinase-activated (PARs):** PAR1, PAR2, PAR4
	**Glycoprotein hormone:** TSH**,** LH	**Relaxin family peptide (RXFP):** RXFP3–RXFP4
	**GPR18** (provisional nomenclature)	**Somatostatin (sst):** sst_1_–sst_5_
	**Histamine:** H_3_, H_4_	**Vasopressin and Oxytocin:** OT
	**Receptors coupled to stimulatory G protein (Gαs)**
	**Orphans:** GPR3-GPR4, GPR6, GPR26, GPR61, GPR65, GPR132, TAAR2, GPR78, GPR174	**Histamine:** H_2_
	**5-Hydroxytryptamine:** 5-HT_4_, 5-HT_6_–5-HT_7_	**Lysophospholipid (LPA):** LPA_3_-LPA_4_
	**Adenosine:** A_2A_, A_2B_	**Sphingosine 1-phosphate (S1P):** S1P_2_–S1P_4_
	**Adrenoceptor (β):** β_1_–β_3_	**Melanocortin:** MC_1_–MC_5_
	**Bile acid**	**Neuropeptide S:** NPS
	**Calcitonin:** CGRP, AM_1_–AM_2_, CT, AMY_1_–AMY_3_,	**Purinergic P2Y:** P2Y_11_
	**Cholecystokinin:** CCK_1_, CCK_2_	**Parathyroid hormone:** PTH_1_–PTH_2_
	**Corticotropin-releasing factor:** CRF_1_, CRF_2_	**Prostanoid:** DP_1_, EP_2_, EP_4_
	**Dopamine:** D_1_, D_5_	**Relaxin family peptide (RXFP):** RXFP1–RXFP2
	**Endothelin:** ET_A_	**Trace Amino:** TA_1_
	**Estrogen (G protein-coupled):** GPER	**Vasopressin and Oxytocin receptors:** V_2_
	**Glucagon:** glucagon, GLP-1–GLP-2, GIP, GHRH, secretin	**Vasoactive intestinal peptide and PACAP:** VPAC_1_, VPAC_2_, PAC_1_
	**Glycoprotein hormone:** FSH, LH	


Based on Alexander et al. (2013).

Assuming the physiological significance of cAMP eﬄux and *extracellular cAMP-adenosine pathway* in modulating GsPCR signaling, impairment of this extracellular feedback mechanism may have profound impact in several physiological processes leading to pathological conditions associated to cardiovascular, muscular and endocrine disorders and/or inflammatory diseases. For example, cAMP secreted from cardiomyocytes attenuates the development of hypertrophy and fibrosis induced by continuous activation of β_2_-adrenoceptors (Sassi et al., 2014). Similarly, cAMP eﬄux induced by prolong stimulation of β_2_-adrenoceptors attenuates skeletal muscle contraction, preserving muscle from deleterious effects of massive Ca^2+^ release from the sarcoplasmic reticulum (Duarte et al., 2012). In pancreatic acinar cells, cAMP egression through MRP4 clearly attenuates the development of acute pancreatitis induced by caerulein (Ventimiglia et al., 2015). On the other hand, inhibition of cAMP eﬄux with probenecid reduces bovine sperm capacitation (Osycka-Salut et al., 2014). Finally, the *extracellular cAMP-adenosine pathway* is able to exert immunoregulatory effects by modulating monocyte function and differentiation through A_2A_/A_2B_ receptors (Sciaraffia et al., 2014), which is consistent with the marked reduction of ecto-5′-nucleotidases expression in lymphocytes from patients with a variety of immunodeficiency diseases (Resta et al., 1998). Interestingly, certain antioxidants derived from plants such as the flavonoid quercetin, have been found to inhibit cAMP eﬄux transporters (Pavan et al., 2015), which open new perspectives in the use of natural products as regulators of extracellular cAMP signaling for therapeutic purposes.

## cAMP Signaling Microdomains

The compartmentalized expression of cAMP signaling components such as GPCR, AC, PKA, and Epacs (Laflamme and Becker, 1999; Zaccolo, 2009; Terrin et al., 2012) allows the establishment of multiple intracellular cAMP subcompartments. For example, PDEs bind to A kinase-anchoring proteins (AKAPs) forming a PKA-PDE-AKAP complex (Stangherlin and Zaccolo, 2011; Terrin et al., 2012). Likewise, specific AC isoforms have been identified as components of AKAP complexes (Dessauer, 2009), contributing to intracellular compartmentalization of cAMP signaling. In fact, localized expression of ACs generate specific microdomains of elevated concentration of cAMP ensuring signaling specificity, such as those observed in the transverse tubule/junctional sarcoplasmic reticulum network of cardiac and skeletal muscle (Gao et al., 1997; Zaccolo et al., 2002; Menezes-Rodrigues et al., 2013).

Interestingly, the localized expression of MRPs may also play an important role in the intra- and extracellular cAMP signaling process (Cheepala et al., 2013). For example, in the gut epithelia signal compartmentalization of cAMP with MRP4 is essential for cAMP-dependent regulation of chloride channel function (Li et al., 2007). As well, contraction of cardiac myocytes induced by activation of β-adrenoceptor/AC/cAMP cascade can be locally regulated by MRP4 (Sellers et al., 2012). In addition, in vascular smooth muscle cells, MRP4 was found in caveolin-rich membrane fractions regulating cAMP/PKA/CREB pathway cascade (Sassi et al., 2008). Thus, multiprotein signaling complexes, which include MRPs may affect the final GsPCR/cAMP-dependent cellular response. Of more importance, the compartmentalized expression of ARs in caveolae/cholesterol-rich microdomains (Sitaraman et al., 2002; Cordeaux et al., 2008; Garg et al., 2009; Assaife-Lopes et al., 2010) suggests that colocalization of MRP4 and ARs in caveolae microdomains coordinates the cAMP eﬄux in the vicinity of the AR domain (Xie et al., 2011; D’ Ambrosi and Volonte, 2013).

On the other hand, activation of ARs involved in the *extracellular cAMP-adenosine pathway* also depends on the availability and distribution of the ecto-enzymes. Ecto-phosphodiesterase and ecto-nucleotidases or adenosine deaminase can very rapidly modulate the extracellular concentrations of adenosine, which will directly affect receptor subtype activation, and as consequence, the final cell response. Interestingly, while all human AR subtypes contain the caveolin binding domain (Mundell and Kelly, 2011), studies analyzing the subcellular distribution of A_1_ receptors on cardiomyocytes showed that selective activation of A_1_ receptors with CCPA results in the switch of A_1_ receptor from caveolin-3-enriched domains to the bulk plasma membrane (Lasley, 2011). The lipid raft localization of A_1_ receptor seems to be essential for the A_1_-dependent activation of K_ATP_ channels that is involved in cardioprotective effects of adenosine. Disruption of cholesterol-rich microdomains with methyl-cyclodextrin drastically reduces the effect of ARs on rat ventricular myocytes K_ATP_ channels (Garg et al., 2009; D’ Ambrosi and Volonte, 2013).

## Conclusion

The existence of a system involved in sequential extrusion and extracellular degradation of cAMP definitively extend the intracellular signaling relevance of cAMP to an extracellular level, which allows paracrine and/or autocrine feedback signaling, depending on the AR subtype expressed on the target and neighboring cells. In view of the recognized ability of adenosine to regulate many cellular processes (Burnstock, 2007), the existence of the *extracellular cAMP-adenosine* reveals new insights into the regulatory mechanisms of cellular response triggered by GsPCR and provides novel therapeutic targets for treatment of a number of diseases associated with dysfunction of GPCR signaling cascade.

## Conflict of Interest Statement

The authors declare that the research was conducted in the absence of any commercial or financial relationships that could be construed as a potential conflict of interest.
